# Expression, transport, and storage of fetuin-B in human granulosa cells

**DOI:** 10.1038/s41598-026-36199-6

**Published:** 2026-01-23

**Authors:** Bartosz Linek, Ann-Christin Meyer, Carmen Schoppe, Thorsten Enklaar, Dirk Prawitt, Walburgis Brenner, Konstantin Hofmann

**Affiliations:** 1 Department of Obstetrics and Gynecology , University Medical Center of Johannes Gutenberg University Mainz , Langenbeckstraße 1, 55121 Mainz, Germany; 2 King’s Fertility Fetal Medicine Research Institute, London, UK; 3 Department of Pediatrics , University Medical Center of Johannes Gutenberg University Mainz , Langenbeckstraße 1, Mainz, 55121 Germany

**Keywords:** Fetuin-B, Granulosa cells, Follicular fluid, Oocyte, Fertilization, Cell biology, Molecular biology, Reproductive biology

## Abstract

Fetuin-B is crucial in fertilization through inhibiting ovastacin, an enzyme that hardens the zona pellucida. Primarily synthesized in the liver, fetuin-B is also found in the placenta, tongue, and follicular fluid produced by granulosa cells. This study investigated fetuin-B localization and production in granulosa cells. Samples from 45 women undergoing intracytoplasmic sperm injection therapy at the University Medical Center Mainz were analyzed. Serum and follicular fluid samples were retrieved during egg collection. Granulosa cells were cultured and sampled at different time points. *FETUB* gene expression was quantified by RT-qPCR, with HepG2 cells as a positive control. Fetuin-B levels in granulosa cells and supernatants were measured by ELISA. Fetuin-B was localized using immunocytochemical staining. HepG2 cells showed strong *FETUB* expression, while granulosa cells exhibited minimal transcription. Fetuin-B levels were highest in serum and significantly lower in follicular fluid (*p* < 0.001). Supernatant fetuin-B peaked on day 1 (*p* = 0.004), while intracellular fetuin-B exceeded extracellular (*p* = 0.008). Immunocytochemistry revealed a largely uniform intracellular distribution, with notable accumulations in filopodia. These findings indicate that granulosa cells store, release, and re-internalize fetuin-B but do not synthesize it. The early release at culture onset suggests a regulatory role in supporting fertilization conditions.

**Supplementary Information:**

The online version contains supplementary material available at 10.1038/s41598-026-36199-6.

## Introduction

Fetuin-B is a plasma protein belonging to the cystatin family of cysteine protease inhibitors and is crucial in oocyte fertilization^1–3^. Although the liver is considered the primary site of fetuin-B synthesis, *FETUB* mRNA has also been found, at lower levels, in the placenta and tongue and has been identified in several mammalian species, including humans, rodents, and bovines^1,2^. Notably, it has also been detected in human follicular fluid, which is produced by granulosa cells.

Fetuin-B enables oocyte fertilization by inhibiting the enzyme ovastacin, which cleaves the zona pellucida protein ZP2, leading to zona pellucida hardening and preventing sperm penetration^4–8^. Thus, ovastacin inhibition is essential for fertilization. Mice lacking the fetuin-B coding gene (*FETUB*-/-) are infertile due to the absence of ovastacin inhibition^9^. In humans, serum fetuin-B levels have been associated with in vitro fertilization (IVF) success rates^10^.

Granulosa cells are the predominant cell type in ovarian follicles^11^. They form a complex with the oocyte and are found exclusively within follicles. They provide nutrients and signaling molecules to the oocyte^2^, maintain the biochemical environment, and secrete follicular fluid into the follicular cavity^12^. Granulosa cells also have endocrine functions, producing estradiol during the follicular phase and progesterone during the luteal phase^13^. Additionally, granulosa cells express follicle-stimulating hormone (FSH) and luteinizing hormone (LH) receptors^14^.

Fetuin-B was detected in granulosa cell culture supernatants^15^. The presence of fetuin-B in granulosa cells and the potential correlation between intracellular concentration and serum fetuin-B concentrations may be relevant to IVF or intracytoplasmic sperm injection (ICSI) outcomes.

These findings suggest a previously unexplored link between systemic and intrafollicular fetuin-B dynamics. The present study therefore aimed to characterize the localization and production of fetuin-B in granulosa cells and their surrounding environment.

## Methods

### Study population and study design

Experimental samples were collected from 45 women undergoing fertility treatment at the University Medical Center Mainz fertility center. As part of a planned ICSI therapy, the participants received controlled ovarian stimulation following an antagonist protocol with FSH alone or in combination with LH together with a gonadotropin-releasing hormone antagonist (Ganirelix). Ovulation was triggered with choriogonadotropin alpha prior to follicle aspiration, during which follicular fluid and oocytes were collected as part of routine clinical procedures.

### Serum and follicular fluid

On the day of follicular puncture, blood was drawn from the patients using a serum tube (Sarstedt, Nürnberg, Germany) and centrifuged (861 × g, 10 min, room temperature). The serum was aliquoted into new microtubes, quickly frozen using liquid nitrogen, and stored at − 20 °C. The follicular fluid was homogenized by pipetting up and down with a serological pipette and aliquoted.

### Extraction of the granulosa cells

Cumulus-oocyte complexes were transferred into 300 µL of hyaluronidase (FertiPro NV, Beernem, Belgium) to remove most of the granulosa cells surrounding the oocytes. The remaining corona radiata cells, which adhere tightly to the oocyte, were mechanically detached using a stripper pipette in a fresh cavity containing 300 µL of flushing medium (FertiPro NV, Beernem, Belgium). The oocytes were then washed twice in 0.5 mL of flushing medium. Granulosa cells were collected directly from the first and second solutions.

### Cell culture of granulosa cells

Cell samples were first centrifuged at 250 × g for 5 min. The supernatant was removed, labeled as “d0,” and frozen. Granulosa cells were cultured in DMEM-F12 Nutrient Mixture (Ham) medium (Gibco Life Technologies Carlsbad, USA) containing 10% fetal calf serum (FCS; Gibco Life Technologies, Carlsbad, USA) and 1% penicillin-streptomycin (Sigma-Aldrich, Merck, Darmstadt, Germany), defined as complete medium. The cells were incubated in a humidified atmosphere at 37 °C and 5% CO_2_. For all functional assays, cells were detached using Accutase (Carl Roth GmbH + Co. KG, Karlsruhe, Germany), seeded in cell culture vessels, and cultured to 80% confluency.

As the cultures developed differently, harvesting was performed on different days. After the first day of cell culture, the supernatant was collected, labeled as “d1,” and frozen. For the evaluation, the supernatants of the cultures with an intermediate medium change were designated as “dx.” As soon as a confluence of approximately 80% was determined by visual inspection, the culture was harvested and aliquoted as “dHarvest.” All cell culture supernatants were stored at − 20 °C.

The cell monolayer was washed with 2.5 to 5 mL Dulbecco’s phosphate-buffered saline (Sigma-Aldrich, Merck, Darmstadt, Germany), and the granulosa cells were detached from the culture vessel with 150 µL Accutase (Carl Roth GmbH + Co. KG, Karlsruhe, Germany). The reaction was stopped by adding 2.85 mL of complete medium, and the suspension was transferred to a 15 mL centrifuge tube; 15 µL of the cell suspension was removed, and the cell count was determined using the LunaTM cell counting slide. Each cell suspension (1 mL) was seeded on slides. The remaining detached cells were centrifuged at 250 × g for 5 min. The supernatant was removed and discarded, and the cell pellet was frozen at − 20 °C.

### Cell culture of HepG2 cells

Since the HepG2 cell line (ATCC, Manassas, USA) is known to express the protein fetuin-B^16^, this was used to create a positive control for *FETUB* expression. HepG2 cells, derived from a human liver cancer, were thawed from cryopreservation and cultured in RPMI 1640 medium (Gibco Life Technologies, Carlsbad, California, USA) with 1% L-glutamine (Gibco Life Technologies, Carlsbad, California, USA), 1% penicillin-streptomycin (Sigma-Aldrich, Merck, Darmstadt, Germany), and 10% FCS (Gibco Life Technologies, Carlsbad, California, USA). Cells were centrifuged, and the pellet was resuspended in medium. Cultures were maintained at 37 °C in a humidified atmosphere with 5% CO₂ and first passaged after 72 h. Cells were harvested at 80% confluency using trypsin (Sigma-Aldrich, Merck, Darmstadt, Germany), centrifuged, and stored as a pellet at − 20 °C.

Before use, 500 µL of lysis buffer (comprising 953.2 mg 20 mM HEPES, Carl Roth GmbH + Co. KG, Karlsruhe, Germany), 2.3376 mg 0.2 M NaCl (Carl Roth GmbH + Co. KG, Karlsruhe, Germany), 60.99 mg 1.5 M MgCl_2_ (Carl Roth GmbH + Co. KG, Karlsruhe, Germany), 29.77 mg 0.4 M EDTA (Sigma-Aldrich, Merck, Darmstadt, Germany), 2 mL 1% Triton X-100 (Merck KGaA, Darmstadt, Germany), 50 µL DL-dithiothreitol (Sigma-Aldrich, Merck, Darmstadt, Germany), 50 µL protease inhibitor (Sigma-Aldrich, Merck, Darmstadt, Germany), 50 µL phosphatase inhibitor (Sigma-Aldrich, Merck, Darmstadt, Germany), and 4,350 µL deionized water were added and the cells were resuspended.

### RNA extraction, reverse transcription, and real-time polymerase chain reaction

RT-qPCR was performed to quantify gene expression. Randomly pooled granulosa cells were collected at two points for RNA isolation: directly from the oocyte washing medium on day 0 (d0) and from the cell culture after 24 h (d1). At d1, the culture medium was removed, and adherent granulosa cells were lysed directly for RNA extraction. In addition to the cDNAs of the granulosa cell pool samples, the 1:10 diluted cDNA of HepG2 cells provided a positive control for each RT-qPCR run. The granulosa cells from the oocyte washing medium were defined as Granulosa Cell Pool 1 (GC-Pool1), while the granulosa cells that had been in cell culture for 24 h were divided into three samples (GC-Pool2, GC-Pool3, and GC-Pool4).

RNA was extracted from granulosa cells and HepG2 cells using the NucleoSpin^®^ RNA Plus Kit (MACHEREY-NAGEL GmbH, Düren, Germany), and the concentration and quality of the RNA were assessed with a NanoDrop One Spectrophotometer (Thermo Scientific, Waltham, MA, USA). The cDNA was synthesized using a master mix containing RiboLock RNase Inhibitor (Thermo Scientific, Vilnius, Lithuania), 10X M-MuLV Reverse Transcriptase Buffer (New England BioLabs Inc., Frankfurt am Main, Germany), Oligo(dT)161028B008D04 (Metabion, Planegg, Germany), M-MuLV Reverse Transcriptase M0253L (New England BioLabs Inc., Frankfurt am Main, Germany), and deoxynucleotide solution mix (New England BioLabs Inc., Frankfurt am Main, Germany).

Four RT-qPCR runs were performed on the granulosa cell pools for the fetuin-B coding gene (*FETUB*), with expression levels normalized to the housekeeping gene beta-actin (*ACTB*), which also verified cDNA integrity and RNA quality. Specific primers for both target genes were designed and validated using Primer-BLAST (NCBI)^17^. Table 1 lists primer sequences. RT-qPCR reactions were performed in 96-well plates using the KAPA SYBR Fast (SIGMA-ALDRICH, MERCK Darmstadt, Germany) on a LightCycler^®^480 II thermocycler (Roche, Basel, Switzerland). F standard curves for *FETUB* and *ACTB* were generated from serial dilutions (1:5, 1:10, 1:20, 1:40, 1:80, 1:160) of HepG2 cDNA and used to calculate sample concentrations. Amplification curves were analyzed using LightCycler^®^480 software (Version 1.5.0.39, Roche, Basel, Switzerland; https://www.roche.com).

The thermocycling conditions were as follows: initial denaturation at 95 °C for 5 min, followed by 45 cycles at 95 °C for 5 s, and primer-specific temperatures of 60 °C for *ACTB* and 61 °C for *FETUB* for 5 s. Crossing points were determined in cycles via fluorescence measurement, and relative expression levels were calculated using the standard curve.

**Table 1:** Primers used for the housekeeping gene beta-actin (ACTB) and the fetuin-B coding gene (FETUB), which were used in RT-qPCR.

Forward primer	Reverse primer
FETUB-201-5/6-F: 5’CTC CGA CTC TGT GCC TGT TG3‘	FETUB-201-7-R: 5’TGC TGG GAT TCT TCC ACC TTG3’
ACTB-F: 5‘GGC ATC CTC ACC CTG AAG TA3’	ACTB-R: 5’GGG GTG TTG AAG GTC TCA AA3‘

### Protein measurements

After adding lysis buffer to the granulosa cells, they were incubated on ice and centrifuged (17,000 × g, 10 min, 4 °C). The amount of intracellular total protein in granulosa cells was determined using the BCA method (Pierce BCA Protein Assay Kit, Thermo Scientific Inc., Waltham, USA) according to the manufacturer’s instructions. Fetuin-B was assayed in triplicate using a commercial ELISA (human fetuin-B DuoSet; R&D Systems, Minneapolis, USA), following the manufacturer’s protocol. Since the fetuin-B concentration was considerably influenced by the volume of added lysis buffer, the fetuin-B concentration in the cytoplasm was also calculated for a more precise indication.

### Western blot

For further analysis, granulosa cells from eight patients undergoing ICSI treatment were collected, pooled into four groups (each comprising two patients), lysed, and processed for Western blotting as described below. Cell lysate samples containing 30 µg of protein each were loaded on a 10% polyacrylamide gel. For orientation, we used the Roti-Mark TRICOLOR (Ref. 8271.1, Carl Roth GmbH + Co. KG, Karlsruhe, Germany) and the MagicMark^®^ XP Standard (Catalog # LC5602, Thermo Scientific, Rockford, USA). A semi-dry blotting system (Mini-Protean^®^ Tetra System, BioRad, Feldkirchen, Germany) was used to blot the proteins on a polyvinylidene difluoride transfer membrane (Ref. 88518, Thermo Scientific, Rockford, USA). The blotted membrane was blocked with 3% nonfat dried milk powder (Ref. A0830, PanReac AppliChem, ITV Reagents, Darmstadt, Germany) in Tris-buffered saline with 0.1% Tween20 (Ref. A4974, PanReac AppliChem, ITV Reagents, Darmstadt, Germany) for 1 h at room temperature. The primary antibody (fetuin-B polyclonal, 1:500 in blocking solution, rabbit IgG, anti-human/mouse/rat, Cat. No. 18052-1-AP, Proteintech, Planegg, Germany) was incubated overnight at 4 °C. The secondary antibody (polyclonal goat anti-rabbit immunoglobulins/HRP, Ref. P0448, Agilent Technologies Singapore, Singapore) was incubated for 1 h. A chemiluminescence signal was generated using Western Lightning^®^ Plus-ECL (Reorder No. NEL105001EA, Revvity, Hamburg, Germany) and detected with FluorChem E (Catalog # 92–14860-00, ProteinSimple, Minneapolis, USA). For the loading control, a glyceraldehyde 3-phosphate dehydrogenase primary antibody (1:1,000 in blocking solution; MA5-15738, Thermo Scientific, Rockford, USA) was incubated overnight at 4 °C, followed by a secondary anti-mouse antibody (1:4,000 in blocking solution; polyclonal rabbit anti-mouse immunoglobulins/HRP, Ref. P0260, Agilent Technologies Singapore, Singapore) for 1 h at room temperature.

### Cytochemical staining

After detachment from the culture vessel, granulosa cells were placed on glass slides and incubated at 37 °C for 24 to 48 h. Following incubation, the slides were visually inspected and fixed with Roti-Histofix. They were then incubated overnight at 4 °C with a primary antibody (fetuin-B Polyklonaler Antikörper 20 µL Rabbit/IgG, anti-human, mouse, rat, Kat-Nr.: 18052-1-AP, Proteintech Planegg, Germany). After washing, the slides were incubated for 30 min at room temperature with a secondary antibody (Dako REAL Envision Detection System Peroxidase/DAB+, contains HRP rabbit/mouse, substrate buffer, DAB+, chromogen [x50], REF: K5007, Dako GmbH Jena, Germany). The antibody-marked slides were stained with a 1:50 dilution of 3,3′-diaminobenzidine (DAB; Dako GmbH Jena, Germany), turning the fetuin-B protein brown. Nuclei were counterstained with hematoxylin (Carl Roth GmbH + Co. KG, Karlsruhe, Germany) for 10 min. After dehydration with ethanol, the slides were mounted using xylol (RanReac AppliChem, Darmstadt, Germany) and Roti-HistoKitt (Carl Roth GmbH + Co. KG, Karlsruhe, Germany).

### Statistical analysis

Descriptive data analysis and diagram generation were performed using Microsoft Excel Version 2406 (Microsoft Corporation, Redmond, USA). All values determined are given as mean values ± standard deviation (SD). The Wilcoxon signed-rank test applied with IBM SPSS Statistics 27 (International Business Machines Corporation [IBM], Armonk, USA) was used to assess equality between related samples. Effect sizes were calculated as r = Z/√N. Correlations were evaluated using the Pearson correlation coefficient (r). A positive r value (*r* > 0) indicated a positive correlation, a negative r value (*r* < 0) a negative correlation, and *r* = 0 denoted no correlation.

### Ethical considerations

The data of the treated individuals were coded using double anonymization to ensure data protection and, if necessary, sample traceability. Each treatment sample was given a letter for the person and a number for the type of sample. The Ethics Committee of the Rhineland-Palatinate Medical Association (Landesärztekammer Rheinland-Pfalz) approved the study (2021–15624), which was conducted in accordance with the Declaration of Helsinki^18^. Informed consent was obtained from all participants, including consent for publication of the collected data.

## Results

### Fetuin-B expression in granulosa cells

*FETUB* mRNA was not detectable in GC-Pools 1–3. Conversely, GC-Pool4 showed melting peaks in two of four qPCR runs, which corresponded to those of the HepG2 positive control and the standard samples, suggesting a possible signal for *FETUB. FETUB* expression in GC-Pool4 was normalized to its own *ACTB* expression to assess the relevance of this signal. The resulting relative expression level was 0.01% of *ACTB*, indicating extremely low expression.

However, this result should be interpreted with caution, as *ACTB*, with its high expression rate, is not an optimal reference point. The weak *FETUB* signal in GC-Pool4 was not reproducible and lacked physiological significance. Overall, the qPCR therefore does not indicate reliable *FETUB* expression in GC-Pool4.

### Fetuin-B protein concentrations

ELISA measurements showed that fetuin-B concentrations were significantly lower in the follicular fluid than in the serum (*p* < 0.001; Z = − 3.46; *r* = 0.87; Table 2).

**Table 2:** Fetuin-B protein concentrations of the participants (blood serum, follicular fluid, oocyte wash medium (d0), and cell culture supernatants (d1, dx, dHarvest)) (r: effect size; SD: standard deviation; Z: standardized Z value). Significant values (p < 0.05) are in bold.

	fetuin-B [ng/mL] Mean ± SD	*p*	Z	*r*
Serum	4.1 ± 1.1	**< 0.001**	−3.46	0.87
Follicular fluid	2.3 ± 0.8			
Oocyte wash medium (d0)	8.17 ± 5.00	**0.004**	−2.67	0.89
Culture supernatant (d1)	0.19 ± 0.06			
Culture supernatant (dx)	0.19 ± 0.03	0.203	−1.36	0.45
Culture supernatant (dHarvest)	0.20 ± 0.04			

The highest fetuin-B concentration was found in the oocyte wash medium (d0), while levels in the cell culture supernatants on the following days were lower and remained constant. From the oocyte wash medium (d0) to day 1 of cell culture (d1), fetuin-B concentrations decreased significantly (*p* = 0.004; Z = − 2.67; *r* = 0.89). No significance was found between the day after the first day of cell culture (dx) and the day of harvest (dHarvest), with a p-value of 0.203 (Z = − 1.36; *r* = 0.45; Table 2).

### Intracellular fetuin-B level in granulosa cells

**Fig. 1:**
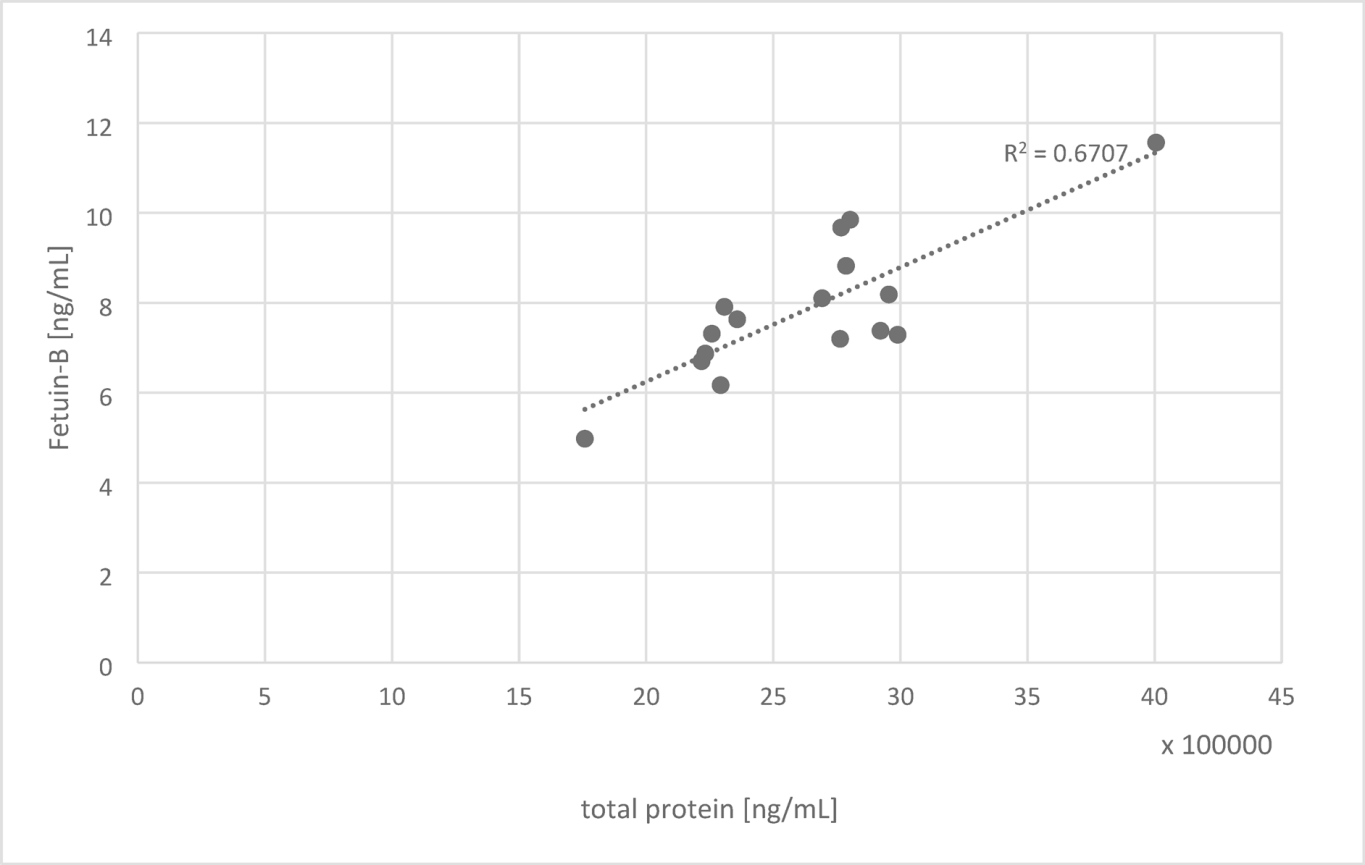
Ratio of intracellular fetuin-B and intracellular total protein in granulosa cells. Each dot represents one person. The dotted line is a linear regression line with a coefficient of determination of 0.67.

Granulosa cell samples contained 7.61 ± 1.22 ng/mL of intracellular fetuin-B. A strong positive correlation was found between total protein and fetuin-B levels (*r* = 0.82; R² = 0.67; Fig. 1). Based on cell count and total protein quantification, the cytoplasmic fetuin-B concentration was estimated at 1.32 µg/mL.

**Fig. 2:**
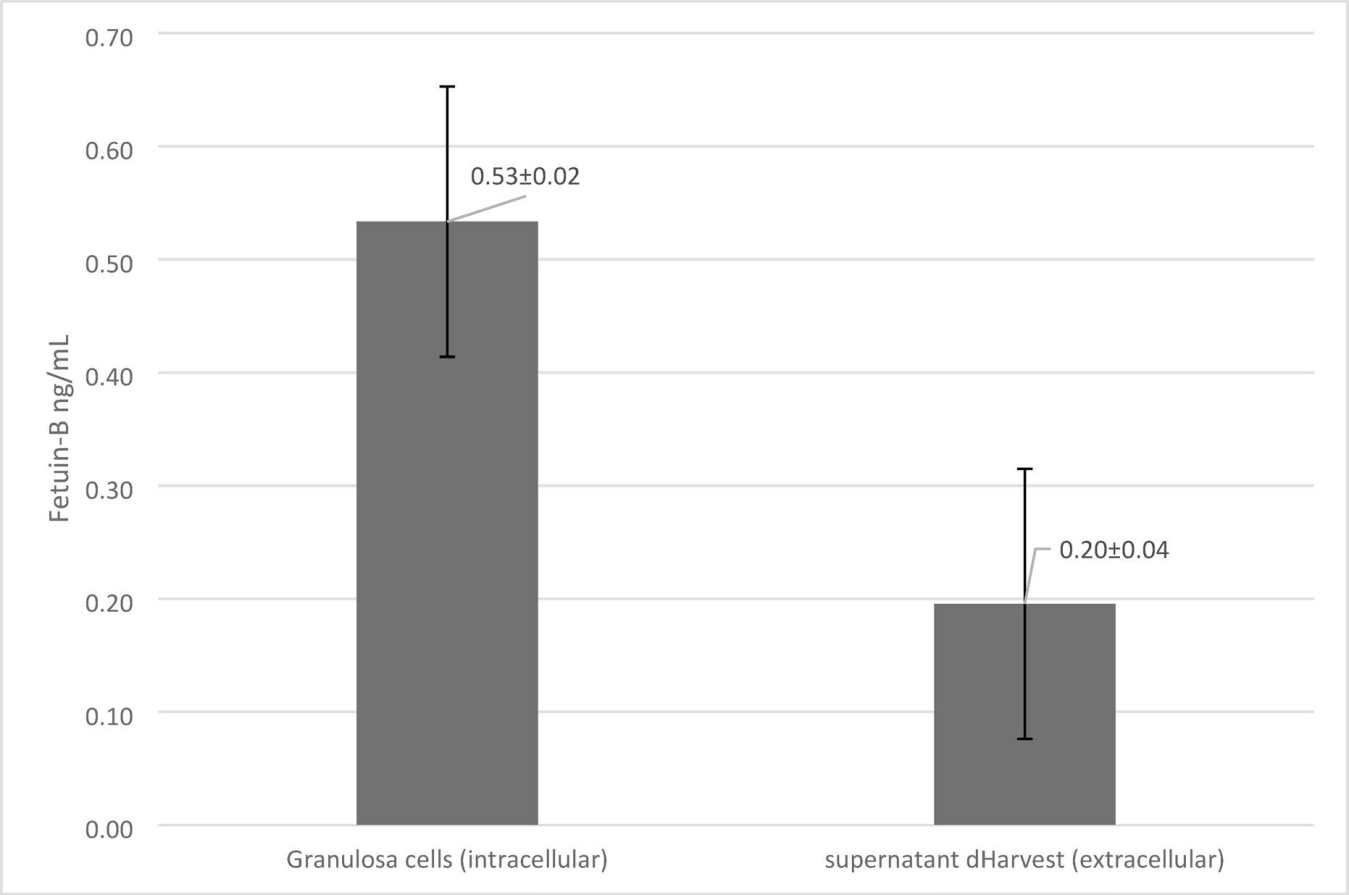
Intra- and extracellular fetuin-B concentrations in granulosa cells and culture medium on the harvest day (dHarvest). Error bars represent standard errors.

Intracellular fetuin-B levels were significantly higher than those in the surrounding culture medium (*p* = 0.008; Z = − 2.67; *r* = 0.89). A weak positive correlation (*r* = 0.21; R² = 0.04) was identified between intracellular fetuin-B in granulosa cells and extracellular concentrations in the culture medium at the harvest (dHarvest; Fig. 2).

**Fig. 3:**
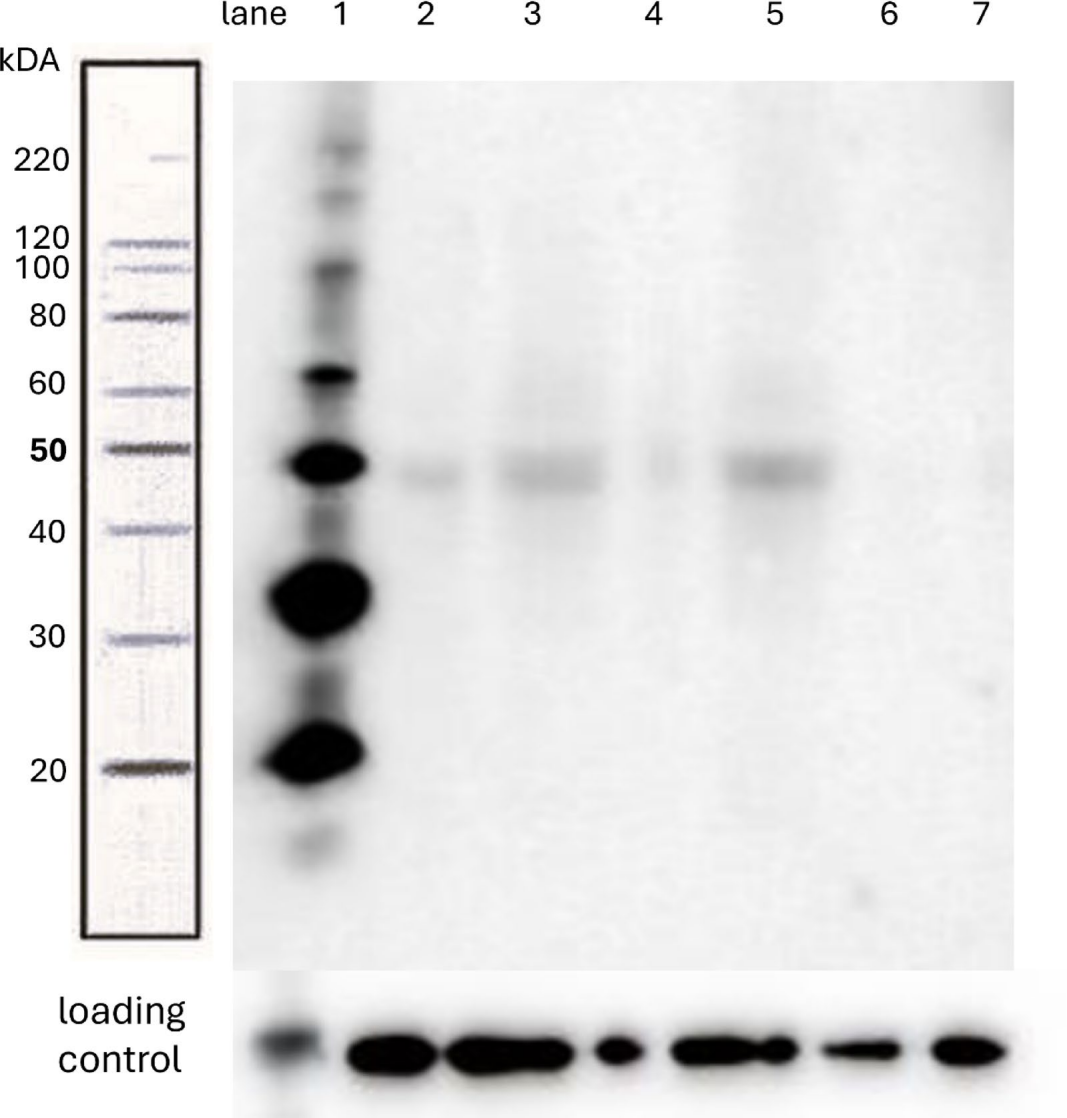
Western-Blot of granulosa cell lysates with fetuin-B primary antibody and GAPDH loading control (at the bottom) lane 1: marker, lane 2–7: cell lysate samples.

We performed Western blot analysis on granulosa cell lysates to confirm the presence of fetuin-B at the protein level. A distinct fetuin-B band was detected in several samples (light signals in lanes 2, 3, and 5 at 50 kDa in Fig. 3), but not in all, consistent with the ELISA-based findings and indicating the presence of intracellular fetuin-B (Fig. 3, full-length blots in Supplementary Fig. S1).

### Cytoplasmic localization of fetuin-B in granulosa cells

**Fig. 4:**
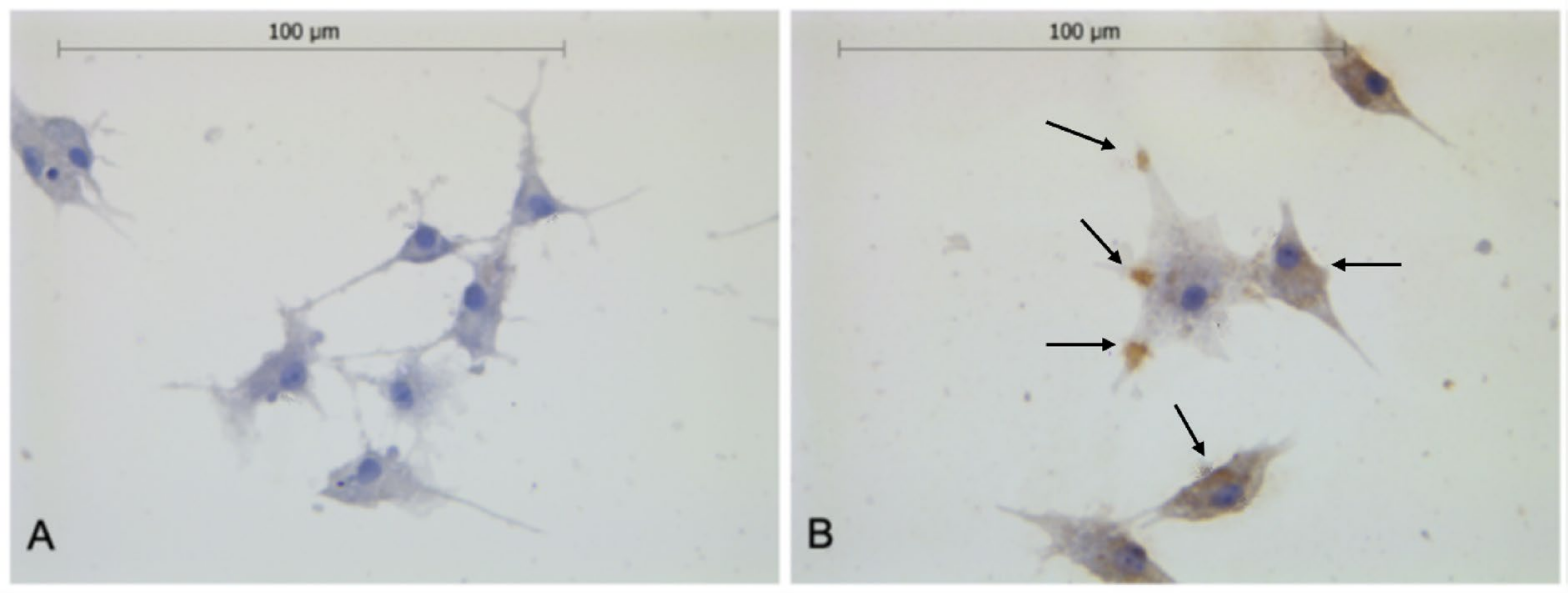
Granulosa cells stained for fetuin-B (**B**) and corresponding negative control (**A**). The negative control (**A**) shows light blue cytoplasmic staining of granulosa cells with dark blue nuclei. In the fetuin-B staining (**B**), similar cellular morphology with distinct brown signals indicating fetuin-B expression (black arrows). The largest central cell with prominent accumulations within the filopodia.

Fetuin-B was detectable in the cytoplasm of granulosa cells cultured on glass slides, determined by immunocytological staining (Fig. 4). An accumulation of fetuin-B in the filopodia was detectable in some cells. Considerable variations in cell morphology were observed between cultures from the different treated individuals.

## Discussion

Fetuin-B has attracted increasing attention in reproductive biology due to its involvement in oocyte maturation and fertilization. Fetuin-B, along with other type 3 glycoproteins, is produced in the liver and has been detected in human testes, placenta, follicular fluid, and the choroid plexus, tissues relevant to fetal development^1,3,9,19^. Granulosa cells, which are integral to follicular development and oocyte support, engage in a range of metabolic and signaling processes crucial for reproductive success. Despite its known presence in reproductive tissues, the specific localization and possible cellular uptake of fetuin-B within granulosa cells have not been demonstrated. Our study therefore provides new insights into fetuin-B biology by investigating its presence and potential origin in human granulosa cells.

Our analysis identified fetuin-B in the granulosa cells, prompting questions about its origin. Using RT-qPCR, we examined whether granulosa cells synthesize fetuin-B. Using RT-qPCR, we found no detectable *FETUB* mRNA expression, indicating that fetuin-B is not endogenously synthesized by granulosa cells.

Høyer et al. found total fetuin in rat granulosa cells and positive results for in situ hybridization of mRNA and RT-qPCR. However, expression and storage levels varied among cells, and fetuin-B was not specifically examined. Therefore, their findings are only partially comparable to ours^20^. Similarly, Yang et al. found no fetuin mRNA in granulosa cells using Northern blot analysis, although fetuin-B was not specifically targeted^21^. Considering these findings, our qPCR results suggest that granulosa cells are unlikely to transcribe fetuin-B, implying that fetuin-B must have been transported from external sources. The exact origin remains uncertain but could include any of the known production sites described above.

Two mechanisms could explain fetuin-B entry into granulosa cells: active uptake or passive diffusion. Based on our data, granulosa cells released the highest amounts of fetuin-B at the beginning of the culture in the oocyte wash medium (d0), with minimal release thereafter. This pattern supports passive diffusion as the most likely mechanism: during oocyte washing, a steep concentration gradient between the granulosa cells and medium promoted fetuin-B release, which declined with the gradient over time.

Granulosa cells can exchange molecules with their environment via diffusion, as shown by the 140 kDa anti-Müllerian hormone^22,23^. This supports the hypothesis that the smaller 55 kDa molecule fetuin-B can also diffuse across the cell membrane^9^. Our results indicated that fetuin-B entered the follicular fluid from the serum and was subsequently taken up by granulosa cells, as evidenced by the absence of *FETUB* mRNA in RT-qPCR analyses.

In our study, fetuin-B concentrations were lower in follicular fluid than in serum. Although fetuin-B is generally assumed to diffuse freely from the serum into the follicular fluid, previous studies reported conflicting data. Floehr et al. and Hu et al. suggested comparable concentrations in serum and follicular fluid, while Bódis et al. found equal levels of the related protein fetuin-A in both compartments^10,24–26^. Conversely, our results revealed a clear difference between fetuin-B concentrations in serum and follicular fluid.

If fetuin-B diffused passively from granulosa cells into the surrounding environment, intracellular and extracellular concentrations should equilibrate. However, this was not observed in our study. The intracellular concentration (1.32 µg/mL) exceeded the extracellular concentration (0.21 ng/mL) in the cell culture medium, although the fetuin-B measured in the supernatant originated from multiple granulosa cells. This disparity could suggest potential active transport or retention mechanisms, although the higher intracellular levels also indicate significant storage within granulosa cells. A weak positive correlation further indicated consistent release patterns across individual cultures. Overall, while passive transport appears more plausible than active secretion, a portion of fetuin-B evidently remained stored within granulosa cells, and further investigation is required.

An important factor in interpreting these results is follicular maturation and participant variability. Spitzer et al. reported variations in protein patterns in follicular fluid between immature and mature follicles, suggesting that the protein profile may depend on the maturation stage of the follicles^27^. Similarly, Fang et al. conducted a larger study involving 182 IVF cycles, finding that while fetuin-B concentrations in serum and follicular fluid correlated, the levels in follicular fluid were lower— consistent with our results. Their study also highlighted the influence of ovarian reserve and follicular stage on fetuin-B levels^28^. Together, these data suggest that fetuin-B concentrations in serum and follicular fluid depend on individual and follicular characteristics.

From a practical perspective, our findings may have implications for IVF procedures. We observed that granulosa cells released the most fetuin-B at the beginning of culture, suggesting that during IVF, the oocyte and its surrounding granulosa cells (corona radiata) should be promptly transferred into fresh medium to preserve sufficient fetuin-B and support fertilization.

Beyond immediate procedural aspects, fetuin-B may function as a biomarker of follicular quality or oocyte maturity. Measuring fetuin-B levels in serum or follicular fluid before IVF/ICSI might help identify cycles with suboptimal follicular conditions. In the long term, modulating fetuin-B concentrations in the culture medium might represent a strategy to improve fertilization outcomes, although biological variability between patients and hormonal influences on granulosa cell behavior must be considered.

During cell culture, considerable variation in cell morphologies was observed between granulosa cell preparations. The observed variations could originate from hormonal influences even before egg retrieval, as the treated individuals were stimulated differently, with combined FSH and LH preparations or only with FSH preparations. It is known that granulosa cells express LH and FSH receptors, which could have enabled LH and FSH to influence the morphology^29^. Differences could also stem from donor age or cellular degeneration^13^. Fetuin-B accumulation in cellular filopodia suggests intracellular movement or redistribution associated with high cell motility (Fig. 4).

**Fig. 5:**
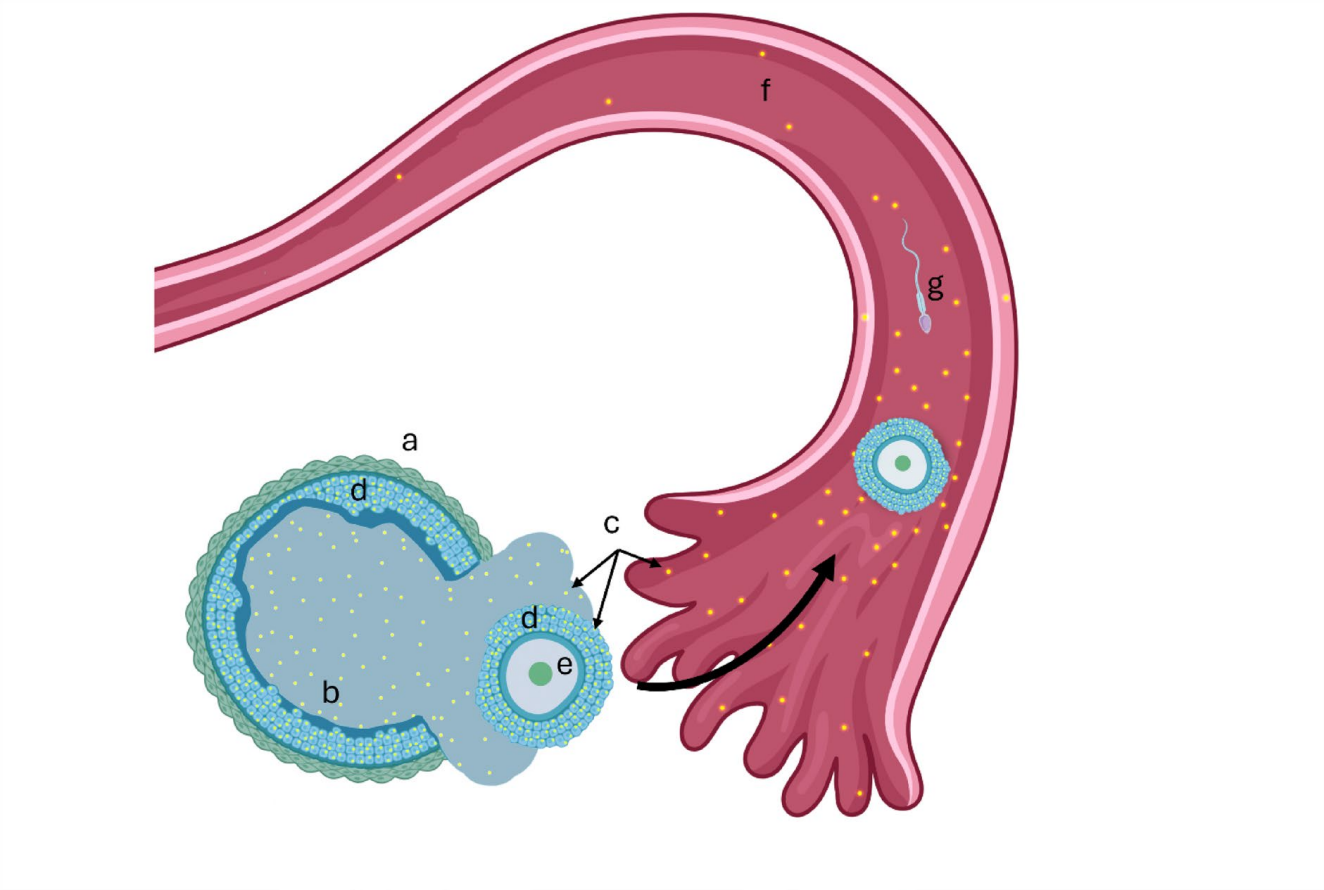
Schematic illustration of the distribution and movement of fetuin-B in the follicle and in the fertilization process; a: follicle; b: follicular fluid; c: fetuin-B; d: granulosa cells; e: oocyte; f: fallopian tube; g: spermatozoon; (Created in BioRender. Hofmann, K. (2025) https://BioRender.com/k62g368).

The localization of fetuin-B in cells adjacent to the oocyte aligns with its proposed physiological role. Cecconi et al. showed that granulosa cells are connected via gap junctions comprising connexin 43 complexes and interact with the oocyte through connexin 37 complexes^30^. The organization of the cumulus matrix surrounding the oocyte is essential for ovulation, oocyte transport, and fertilization^31^. The close proximity may enable fetuin-B to inhibit ovastacin directly, maintaining zona pellucida permeability and facilitating sperm penetration. After ovulation, corona radiata granulosa cells may carry fetuin-B via the fimbria into the fallopian tube, where it can inhibit ovastacin and support fertilization. Fetuin-B is also produced by the tube and thus supports physiological fertilization (Fig. 5)^24^.

### Limitations

A limitation of this study is the small sample size, which reduces the statistical power and limits the generalizability of the findings. Patient populations described in this and previous studies are heterogeneous regarding underlying conditions and indications for assisted fertilization, and differences in hormonal stimulation protocols may have influenced the results. Additionally, it is notable that individuals undergoing fertility treatment are often older than those conceiving naturally^32^. A higher age of the probands could introduce age-related confounding factors, potentially limiting the applicability of the results to younger populations.

Since the cell culture method does not represent a natural environment for the granulosa cells, cell degeneration may occur during cultivation. The morphological differences observed may also reflect a mixture of undifferentiated and differentiated granulosa cells. Our findings suggest that granulosa cells may function as a reservoir for fetuin-B.

Future studies should clarify whether and how hormonal stimulation influences the behavior of granulosa cells. The cells could be subjected to an artificial menstrual cycle to check whether they release or absorb the protein only in a specific cycle phase. Investigating the observed accumulation in the filopodia and its potential hormonal regulation could provide further insights.

## Conclusions

This study identifies granulosa cells as key players in the storage, release, and uptake of fetuin-B but not its synthesis, revealing a previously unrecognized aspect of fetuin-B regulation within the ovarian environment. Granulosa cells released the highest levels of fetuin-B at the start of the culture, which could consequently support oocyte fertilization in a natural environment.

## Supplementary Information

Below is the link to the electronic supplementary material.

Supplementary Material 1

## Data Availability

The datasets used and/or analyzed during the current study are available from the corresponding author on reasonable request.
