# Supplementary material for: Expression, transport, and storage of fetuin-B in human granulosa cells
Source: Sci Rep. 2026 Jan 23;16:3264. doi: 10.1038/s41598-026-36199-6 (PMC12834975; doi:10.1038/s41598-026-36199-6)
Supplement: Supplementary file 1 — Supplementary Material 1 [file 41598_2026_36199_MOESM1_ESM.docx]

**Supplementary Material**


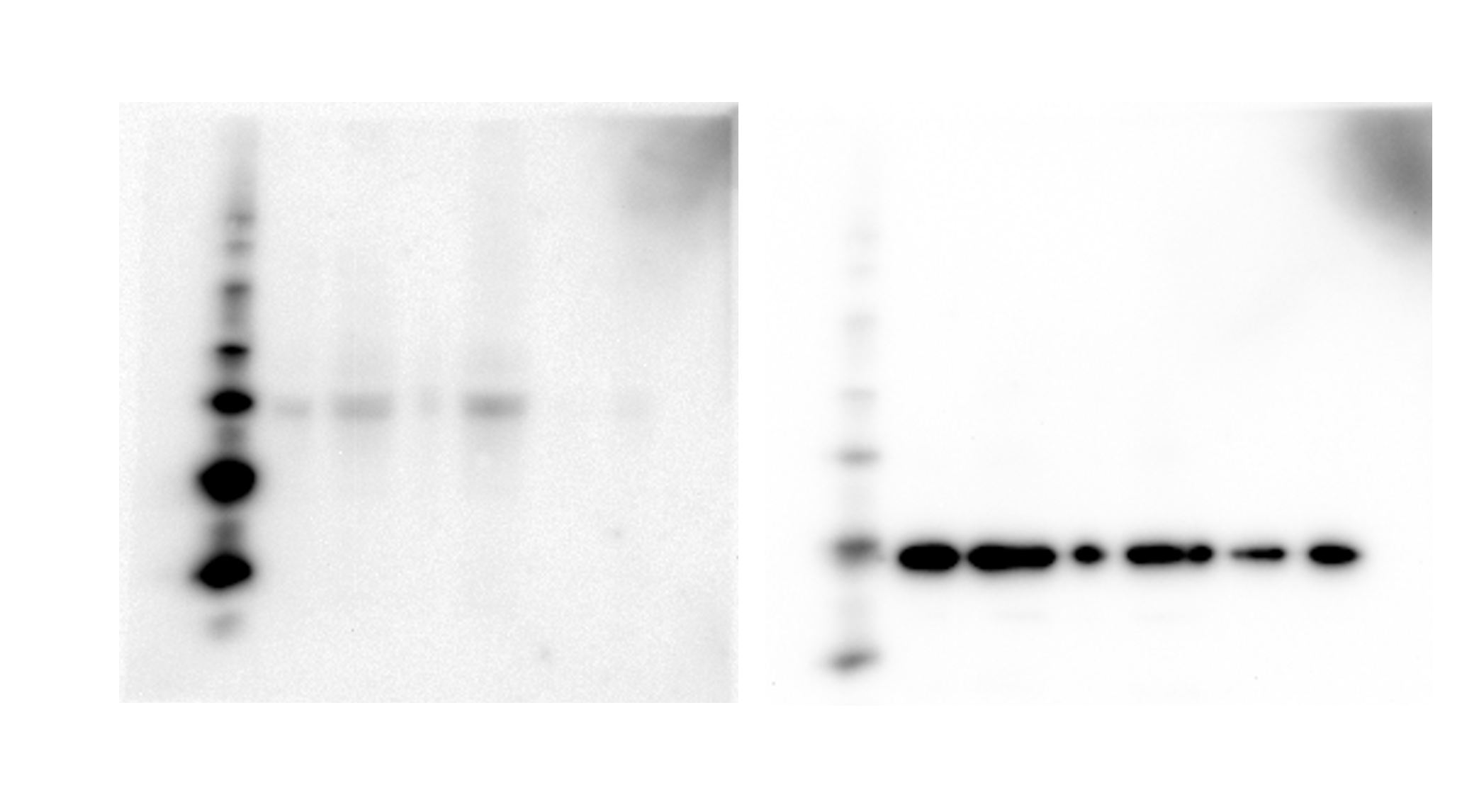


Supp.Fig. S1: Uncropped, full-length Western blot images showing fetuin-B detection in granulosa cell lysates. Membrane edges are visible. The corresponding cropped versions are shown in Figure 3 of the main manuscript.
